# Differential Modulation of WNT/β‐Catenin Pathway‐Associated Molecular Responses by Palmitate and Oleate in Lipid‐Loaded HepG2 Cells

**DOI:** 10.1002/cnr2.70703

**Published:** 2026-10-12

**Authors:** Moayed Al‐Haeri, Yaser Mohammadi, Roghiyeh Pashaei‐Asl, Elham Bahreini

**Affiliations:** ^1^ Department of Biochemistry, Faculty of Medicine Iran University of Medical Sciences Tehran Iran; ^2^ Student Research Committee Iran University of Medical Sciences Tehran Iran; ^3^ Interdisciplinary Research Development Center Iran University of Medical Sciences Tehran Iran

**Keywords:** hepatocellular carcinoma, HepG2 cells, oleate, palmitate, steatosis, WNT/β‐catenin signaling

## Abstract

**Background:**

Palmitate (PA) and oleate (OA), the predominant saturated and monounsaturated fatty acids, respectively, may differentially influence hepatic signaling. However, their individual and combined effects on WNT/β‐catenin‐related responses in HepG2 cells remain incompletely characterized.

**Methods:**

Human HepG2 cells were exposed to PA, OA, or a combined PA/OA treatment for 24 h. Cell viability was determined via MTT assay, while intracellular lipid droplet (LD) accumulation was evaluated using Oil Red O staining. Transcriptional alterations of canonical WNT/β‐catenin cascade genes (WNT, CTNNB1, GSK‐3β, TCF, LEF, Axin1, and Axin2) were analyzed by RT‐qPCR. Protein expression levels of WNT, β‐catenin, and Glypican‐3 (GPC3) were evaluated to assess pathway activation.

**Results:**

Both fatty acids induced dose‐dependent cytotoxicity and intracellular lipid accumulation, with the combined PA/OA treatment provoking the highest LD content. At the molecular level, palmitate predominantly activated the canonical cascade, significantly upregulating WNT, CTNNB1, TCF, and Axin2 mRNA, alongside elevated WNT, β‐catenin, and GPC3 protein levels. Conversely, oleate selectively upregulated LEF and GSK‐3β without coordinated canonical cascade activation, maintaining lower WNT and GPC3 protein expressions. Interestingly, co‐treatment with PA/OA yielded intermediate molecular responses, partially attenuating palmitate‐induced activation of WNT and Axin2, and facilitating a partial recovery of Axin1 expression.

**Conclusion:**

PA and OA differentially modulated WNT/β‐catenin pathway‐associated responses in lipid‐loaded HepG2 cells. PA elicited a broader response involving WNT, CTNNB1, TCF, Axin2, WNT, and β‐catenin, whereas OA produced a more selective response. Their combination partially attenuated PA‐associated changes, highlighting fatty acid composition as a potential determinant of cellular responses to lipotoxic stress.

## 1 Introduction

Over the past decades, profound changes in lifestyle and dietary habits have contributed substantially to the global rise in metabolic disorders. Increased consumption of energy‐dense and high‐fat foods, together with reduced physical activity, can promote lipid accumulation and metabolic disturbances. Experimental and epidemiological evidence indicates that high‐fat dietary exposure can alter hepatic lipid metabolism and contribute to hepatic lipid deposition and metabolic dysfunction [1, 2, 3]. Importantly, the metabolic effects of dietary fat depend not only on the amount consumed but also on fatty acid composition [4]. Western dietary patterns are typically characterized by relatively high intakes of saturated fatty acids, whereas Mediterranean‐type dietary patterns provide greater amounts of monounsaturated and polyunsaturated fatty acids [1, 5]. Differences in dietary fat composition have been associated with variations in metabolic outcomes, including obesity, insulin resistance, Type 2 diabetes mellitus, and chronic liver disease [6]. Beyond their role as energy substrates, fatty acids also act as bioactive molecules that can modulate cellular processes involved in metabolism, inflammation, and stress responses [7].

Non‐alcoholic fatty liver disease (NAFLD), recently redefined as metabolic dysfunction‐associated steatotic liver disease (MASLD), is currently the most prevalent chronic liver disease worldwide and is associated with an increased risk of hepatocellular carcinoma (HCC) [8]. MASLD encompasses a spectrum ranging from hepatic steatosis and steatohepatitis to fibrosis, cirrhosis, and, in some patients, HCC [9]. Disease progression is accompanied by excessive lipid accumulation, oxidative stress, chronic inflammation, mitochondrial dysfunction, and metabolic disturbances that can impair hepatic homeostasis [10, 11]. Lipid overload promotes the production of pro‐inflammatory cytokines and reactive oxygen species, contributing to oxidative stress and lipid peroxidation that can impair cellular macromolecules and membrane integrity [12, 13]. The resulting oxidative imbalance may further amplify cellular injury and disrupt redox‐sensitive signaling processes, thereby affecting pathways involved in cell survival and homeostasis. Persistent oxidative and inflammatory stress may also contribute to DNA damage and genomic instability, providing a mechanistic link between metabolic stress and altered cellular signaling [14, 15]. Thus, metabolic stress associated with hepatic lipid overload may have consequences that extend beyond steatosis itself, potentially influencing signaling networks involved in cellular proliferation, survival, and other processes relevant to hepatocellular tumor biology. Accordingly, defining how specific fatty acids alter cancer‐relevant signaling pathways may provide additional insight into the molecular interface between metabolic liver disease and HCC [16, 17].

Among the signaling pathways involved in hepatic homeostasis and liver cancer, the canonical WNT/β‐catenin pathway has received considerable attention [18]. This evolutionarily conserved pathway regulates cell proliferation, differentiation, migration, and survival. Under basal conditions, cytoplasmic β‐catenin is regulated by a destruction complex containing APC, Axin, and GSK‐3β, which promotes its phosphorylation and subsequent degradation. WNT ligand stimulation inhibits this process, resulting in β‐catenin accumulation and its subsequent translocation to the nucleus, where it interacts with TCF/LEF transcription factors to regulate target‐gene expression [18, 19]. Dysregulation of WNT/β‐catenin signaling has been reported in HCC and is implicated in mechanisms that govern tumor development and progression [20]. Because this pathway integrates signals controlling proliferation, survival, and cellular differentiation, alterations in its activity may represent an important molecular link between metabolic stress and cancer‐associated cellular phenotypes in the liver. In HCC, therefore, changes in β‐catenin abundance or in the expression of pathway‐associated components may be biologically relevant even when they do not by themselves establish a direct carcinogenic effect [17, 21].

Among fatty acids, palmitate (PA) and oleate (OA) are of particular interest because they differ in saturation and can exert distinct cellular effects. PA, a saturated fatty acid, has been associated with lipid accumulation, lipotoxic stress, oxidative stress, inflammation, and metabolic dysfunction [22, 23]. OA, a monounsaturated fatty acid, is generally associated with greater metabolic tolerance and can promote lipid storage in relatively less toxic forms and mitigate some cellular effects of saturated fatty acids [24, 25]. These observations, however, largely derive from metabolic, animal, or cellular models and may not necessarily apply to HCC‐derived cells. Importantly, both PA and OA have also been reported to influence intracellular signaling pathways relevant to cell survival and cancer biology, including components of WNT/β‐catenin signaling [26]. Experimental studies have suggested that PA can affect β‐catenin‐related signaling and the expression of pathway‐associated components [26, 27], whereas the effects of OA appear more context‐dependent, with reports of both protective effects and modulation of β‐catenin‐related responses [28, 29]. The differential effects of saturated and monounsaturated fatty acids are therefore of potential relevance not only to hepatic lipid handling but also to the signaling environment of HCC cells, particularly when metabolic stress intersects with pathways implicated in tumor biology [21].

A further limitation of the existing literature is that PA and OA are frequently examined separately, despite the simultaneous exposure of hepatic cells to multiple fatty acids in vivo [29, 30]. Studies using isolated fatty acids can therefore provide information about individual cellular responses but may not fully capture the molecular effects of combined fatty acid exposure. Moreover, evidence from dietary studies, non‐transformed hepatocyte models, and HCC‐derived cell lines represents distinct biological contexts and should not be considered interchangeable. Consequently, the effects of PA and OA on WNT/β‐catenin pathway‐associated molecular responses may depend on both fatty acid composition and cellular context. However, whether PA and OA produce distinct or interacting molecular responses in lipid‐loaded HepG2 cells remains insufficiently characterized. In particular, the molecular response to combined PA/OA exposure and its relationship to the responses elicited by each fatty acid individually remain unclear.

HepG2 cells were selected as an established human HCC‐derived in vitro model to investigate fatty acid‐induced molecular responses in a hepatic cancer‐cell context. Although HepG2 cells do not represent normal hepatocytes and cannot model the initiation of hepatocarcinogenesis, they provide a reproducible system for examining cellular responses to lipid exposure and changes in signaling‐related molecular markers. Importantly, the use of an HCC‐derived model allows the present study to examine whether fatty acid‐induced metabolic stress is accompanied by differential modulation of molecular components of a signaling pathway with established relevance to HCC biology. This approach does not assume that fatty acid exposure directly causes malignant transformation; rather, it enables characterization of cancer‐relevant molecular responses within an established HCC cellular context [31].

Accordingly, the present study investigated the effects of PA, OA, and their combination on lipid accumulation and selected molecular components associated with canonical WNT/β‐catenin signaling in HepG2 cells. By comparing individual and combined fatty acid treatments, this study aimed to characterize fatty acid‐specific molecular responses under in vitro lipid‐loading conditions and to determine whether combined exposure produces a response distinct from that induced by either fatty acid alone. The findings are interpreted within the context of an HCC‐derived cellular model and are intended to characterize fatty acid‐specific molecular responses rather than to establish mechanisms of hepatocarcinogenic initiation or clinical effects.

## 2 Materials and Methods

### 2.1 Chemicals and Reagents

The materials and reagents utilized throughout the present investigation included oleic acid (Sigma‐Aldrich, Cat. No. 311111) and palmitic acid (Sigma‐Aldrich, Cat. No. P5177). Cell culture experiments were performed using DMEM/F12 medium supplemented with GlutaMAX (Bio Idea Co., Cat. No. BI‐1011), while antibiotic protection was provided by a penicillin–streptomycin solution (Bio Idea Co., Cat. No. BI‐1203). Cell detachment procedures were carried out using a 10× trypsin–EDTA solution (Bio Idea Co., Cat. No. BI‐1602). Dimethyl sulfoxide (DMSO; Cat. No. 102952) served as the solvent where required. Fetal bovine serum (FBS; Biosera Co., Cat. No. F‐1001/100) was used as a culture supplement.

For cell viability assessment, 3‐(4,5‐dimethylthiazol‐2‐yl)‐2,5‐diphenyltetrazolium bromide (MTT; Sigma‐Aldrich, Cat. No. M5655) was employed. Intracellular lipid accumulation was evaluated using Oil Red O powder (Sigma‐Aldrich, Cat. No. 1320‐06‐5). Gene expression analyses were conducted using Super SYBR Green qPCR Master Mix (Yekta Tajhiz Co., Cat. No. YT2552), whereas total RNA extraction and complementary DNA synthesis were performed using the Total RNA Isolation Kit (Yekta Tajhiz Co., Cat. No. FAPDE050) and the cDNA Synthesis Kit (Yekta Tajhiz Co., Cat. No. YT4500), respectively.

### 2.2 Cell Culture

Human HepG2 cells (Cell No. C10096) were obtained from the Iranian Biological Resource Center (IBRC, Tehran, Iran), a certified biological resource repository. The identity of the cell line was verified by the provider prior to distribution, and no additional authentication was performed in our laboratory before experimentation. HepG2 is a well‐established human HCC cell line that is widely used as an in vitro model for investigating hepatic lipid metabolism, lipotoxicity, and steatosis because it retains several hepatocyte‐specific metabolic functions and exhibits a robust capacity for intracellular lipid accumulation.

Cells were cultured in Dulbecco's Modified Eagle Medium/Nutrient Mixture F‐12 (DMEM/F‐12) supplemented with 10% fetal bovine serum (FBS) and 1% penicillin–streptomycin and maintained at 37°C in a humidified atmosphere containing 5% CO_2_. The cells were confirmed to be free of mycoplasma contamination before the initiation of the experiments. For all experiments, cells were seeded into appropriate tissue culture plates and allowed to reach approximately 70%–80% confluence before treatment. The culture medium was renewed every 48 h, and only cultures exhibiting a cell viability exceeding 90%, as determined before experimental treatment, were included in subsequent analyses.

### 2.3 Determination of Optimal PA and OA Concentrations for Lipid Loading in HepG2 Cells

#### 2.3.1 Preparation of Palmitate and Oleate Solutions

Palmitic acid (PA) and oleic acid (OA) stock solutions were prepared separately immediately before cell treatment. For PA, 7.7 mg of palmitic acid was dissolved in 1 mL of 50% (v/v) ethanol to obtain a 30 mM stock solution. The PA solution was incubated in a water bath at 60°C for 5 min to facilitate dissolution. For OA, 8.5 mg of oleic acid was dissolved in 1 mL of absolute ethanol to obtain a 30 mM stock solution. The prepared solutions were transferred to a sterile working environment, and each stock solution was then mixed with an equal volume of culture medium to obtain a 15 mM intermediate solution. These intermediate solutions were subsequently diluted with culture medium to obtain the final experimental concentrations immediately before cell treatment. The fatty acids were therefore administered from ethanol‐based stocks without prior conjugation to fatty‐acid‐free bovine serum albumin (BSA). The same culture medium used for routine HepG2 cell maintenance was used for the final treatment dilutions. The final ethanol concentration corresponding to each treatment was maintained at the same level across the experimental conditions and control groups.

#### 2.3.2 MTT Assay

To establish suitable concentrations of PA and OA for the induction of lipid accumulation, a two‐step dose‐selection strategy was employed using the MTT assay. PA and OA stock solutions were prepared in ethanol and subsequently diluted in culture medium to obtain the desired working concentrations. In all experiments, the final ethanol concentration was maintained below 0.1% (v/v) to minimize solvent‐related cytotoxicity. Vehicle controls containing equivalent amounts of ethanol were included accordingly.

Initially, HepG2 cells were exposed separately to increasing concentrations of PA or OA (0.4–1.2 mM) for 24 and 48 h. Cell viability data obtained from these experiments were used to identify concentrations associated with moderate cytotoxicity and to select an appropriate exposure period. Based on these preliminary evaluations, a 24‐h incubation time was chosen for subsequent studies. In the second phase, PA and OA concentrations producing comparable effects on cell viability were combined to generate a lipid‐loading model. MTT measurements were performed for PA, OA, and PA/OA treatments using two independent biological replicates derived from two separate HepG2 cell cultures on two different days. Within each biological replicate, four technical replicate wells were used for each concentration/treatment condition. The four technical replicates were averaged within each biological replicate and were not considered independent observations for statistical analysis. The resulting values from the two independent biological replicates were used for statistical analysis. IC_50_ values were calculated based on the dose–response data.

For viability assessment, HepG2 cells were seeded in 96‐well plates at a density of 2.5 × 10^4^ cells per well and treated as described above. Following the incubation period, cells were exposed to MTT solution (5 mg/mL) diluted in serum‐free medium and incubated for 4 h at 37°C in a 5% CO_2_ atmosphere. The resulting formazan crystals were dissolved in DMSO, and absorbance was measured at 570 nm using a microplate reader. Cell viability was expressed as a percentage of the vehicle control group according to the following equation:

$$\text{Relative}\;\mathrm{ORO}\;\text{signal}\;\left(\%\right)=\frac{{\mathrm{OD}}_{\text{treated}}}{{\mathrm{OD}}_{\text{control}}}\times 100$$

The concentration required to reduce cell viability by 50% (IC_50_) was determined for each treatment condition.

### 2.4 Semi‐Quantitative Assessment of Lipid Droplet Accumulation Using Oil Red O Assay

Lipid droplet (LD) accumulation was evaluated to assess intracellular neutral lipid deposition following fatty acid treatment in HepG2 cells. Oil Red O (ORO) staining was used as a lipid‐specific method for the qualitative visualization and semi‐quantitative assessment of neutral lipid accumulation.

For this purpose, cells were fixed with 10% neutral buffered formalin to preserve cellular morphology and intracellular lipid droplets. Following fixation, cells were briefly treated with isopropanol before staining. The Oil Red O working solution was prepared from an isopropanol‐based stock solution and filtered prior to use to ensure uniform staining. Cells were subsequently incubated with the ORO working solution at room temperature to allow staining of intracellular neutral lipid droplets. Excess stain was removed by washing with distilled water. The stained lipid droplets were visualized by light microscopy at 40× magnification, and representative images were captured for qualitative assessment of lipid accumulation.

For semi‐quantitative assessment, the ORO retained by the stained cells was extracted using a defined volume of absolute isopropanol, and the absorbance of the extracted dye was measured at 500 nm using a microplate reader. The resulting absorbance was used as a relative index of ORO staining and, consequently, of neutral lipid accumulation under the experimental conditions. Because ORO staining and extraction provide a semi‐quantitative rather than an absolute measurement of intracellular lipid mass, the results were interpreted as relative ORO staining rather than absolute lipid content per cell.

To account for differences in the metabolic/viability status of cells following treatment, the ORO staining signal was additionally expressed relative to the corresponding control condition and adjusted using the MTT‐derived metabolic activity/viability. Importantly, this adjustment was not considered a direct normalization to cell number or cellular mass. The resulting value was therefore interpreted as a viability‐adjusted ORO index rather than lipid content per cell. The index was calculated as follows:

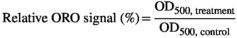

$$\text{Viability}-\text{adjusted}\;\mathrm{ORO}\;\text{index}=\frac{\text{Relative}\;\mathrm{ORO}\;\text{signal}}{\text{Relative}\;\mathrm{MTT}\;\text{activity}}$$

where relative ORO signal represents the ORO absorbance expressed relative to the corresponding control group, and relative MTT activity represents the MTT‐derived metabolic activity expressed relative to the corresponding control group (control = 100%).

### 2.5 RT‐qPCR Analysis of WNT/β‐Catenin Signaling Genes

RT‐qPCR was performed to investigate transcriptional changes in key components of the WNT/β‐catenin signaling pathway, including WNT ligands, β‐catenin (CTNNB1), glycogen synthase kinase‐3β (GSK‐3β), TCF/LEF transcription factors, and the downstream target genes Axin1 and Axin2, in HepG2 cells following 24 h exposure to PA, OA, and PA/OA.

Total RNA was isolated from treated cells using a commercial RNA extraction kit according to the manufacturer's instructions, with the addition of 2‐mercaptoethanol to minimize RNase‐mediated RNA degradation. RNA concentration and purity were assessed using a NanoDrop spectrophotometer, and RNA integrity was further evaluated by agarose gel electrophoresis. Equal amounts of RNA (1 μg) were used for first‐strand cDNA synthesis using a reverse transcription kit (Yekta Tajhiz, Iran). The reverse transcription reaction was performed in a final volume of 20 μL, and the resulting cDNA was used directly for qPCR without further dilution.

Quantitative PCR was performed using SYBR Green chemistry on an ABI 7500 Fast Real‐Time PCR System. Gene‐specific primers were designed using Primer‐BLAST (Table 1). Each reaction was performed in a final volume of 10 μL containing 1 μL of cDNA. Amplification was initiated with an initial denaturation at 95°C for 10 min, followed by 40 cycles of denaturation at 95°C for 15 s, annealing at the primer‐specific melting temperature (Tm) for 30 s, and extension at 72°C for 30 s. No‐template controls (NTCs) and no‐reverse‐transcriptase (no‐RT) controls were included to monitor potential contamination and genomic DNA amplification, respectively. Melting‐curve analysis was performed at the end of each run, and a single peak was observed for each target, supporting amplification specificity.

**TABLE 1:** Primer sequences used for RT‐qPCR.

Genes	Direction	Sequence (5′→3′)	PCR‐Tm °C
TCF	F	5′‐TTGACCGACAGACTTTATGGTGC‐3′	61
	R	5′‐CTGAAGAGGGTGGGCTGAGG‐3′	
LEF	F	5′‐TCAGTCATCCCGAAGAGGAAGG‐3′	61
	R	5′‐TGAGAGGTTTGTGCTTGTCTGG‐3′	
GSK3B	F	5′‐AGGCTACCATCCTTATTCCTCCTC‐3′	64
	R	5′‐AGGTCTGTCCACGGTCTCCAG‐3′	
AXIN1	F	5′‐TGTGAGGAGGGAGAACTGGTGTC‐3′	65
	R	5′‐AGTATGGTGGGGTGGGGGAGG‐3′	
AXIN2	F	5′‐TGAAGCCTGCCACCAAGACC‐3′	61
	R	5′‐CATTTTCCTCCATCACCGACTG‐3′	
TBP	F	5′‐GTTCAGCAGTCAACGTCCCAG‐3′	62
	R	5′‐GTCATGGGGGAGGGATACAGTG‐3′	

TBP (TATA‐box binding protein) was used as the internal reference gene for normalization [17]. Relative gene expression levels were calculated using the 2^−ΔΔCt^ method, assuming 100% amplification efficiency. Data are presented from 2 to 4 independent experiments.

### 2.6 Western Blot Analysis

Western blotting was performed to evaluate WNT, β‐catenin, and Glypican‐3 (GPC3) expression in treated HepG2 and Huh7 cells, with β‐actin as the loading control. Cells grown in 6‐well plates were lysed in buffer containing 1% SDS, 10% glycerol, 50 mM Tris–HCl (pH 6.8), and protease inhibitor cocktail. Lysates were incubated on ice for 15 min and centrifuged at 13,000 rpm for 10 min at 4°C, and the supernatants were collected. Protein concentrations were determined using the Bradford assay, and 30–40 μg of protein per sample was separated by 10% SDS‐PAGE and transferred to PVDF membranes using wet or semi‐dry transfer.

Membranes were blocked with 5% nonfat dry milk in TBS‐T (0.1% Tween‐20) for 1 h at room temperature and incubated overnight at 4°C with rabbit anti‐β‐catenin (Cat. No. 9562S, Cell Signaling Technology; 1:1000), rabbit anti‐WNT (Cat. No. 27935‐1‐AP, Proteintech; 1:1000), rabbit anti‐GPC3 (Cat. No. ab207080, Abcam; 1:1000), or rabbit anti‐β‐actin (Cat. No. ab8227, Abcam; 1:2500). Following TBS‐T washing, membranes were incubated with goat anti‐rabbit IgG H&L‐HRP (Cat. No. ab6721, Abcam; 1:10 000) for 1 h at room temperature. Protein bands were visualized using ECL and a gel documentation system. The approximate molecular weights were 92 kDa for β‐catenin, 44 kDa for WNT, 66 kDa for GPC3, and 42 kDa for β‐actin. Band intensities were quantified using ImageJ, normalized to β‐actin, and expressed relative to the control group. Three independent biological replicates were analyzed (*n* = 3).

### 2.7 Statistical Analysis

Experiments were performed using biological and technical replicates, as indicated for each experiment. Where applicable, three technical replicates were obtained from each of two independent biological replicates within an experimental run. Data are presented as mean ± standard deviation (SD). Statistical analyses were performed using one‐way analysis of variance (ANOVA), followed by an appropriate post hoc test when applicable. The assumptions of normality and homogeneity of variances were assessed before performing ANOVA. A *p*‐value < 0.05 was considered statistically significant.

## 3 Results

### 3.1 Investigation of the Cytotoxic Effects of Palmitate, Oleate, and Their Combination on HepG2 Cell Viability

The cytotoxic effects of palmitate and oleate on HepG2 cells were evaluated using the MTT assay after 24 and 48 h of treatment. Cell viability decreased in a concentration‐dependent manner as the concentrations of both fatty acids increased. For palmitate, the estimated IC_50_ values were approximately 0.8 mM after 24 h and 0.7 mM after 48 h of treatment. Similarly, oleate reduced cell viability in a concentration‐dependent manner, with estimated IC_50_ values of approximately 1.0 and 0.9 mM after 24 and 48 h, respectively. Since no significant difference was observed between the IC_50_ values obtained at 24 and 48 h (*p* > 0.05), the 24‐h incubation period was selected for subsequent experiments. The IC_50_ concentrations were used as standardized, experimentally defined exposure levels associated with a measurable cellular response and were not intended to represent physiological fatty‐acid concentrations.

To establish an appropriate combined PA/OA treatment condition, the individual IC_50_ concentrations of PA (0.8 mM) and OA (1.0 mM) were initially combined. However, this combination resulted in approximately 20% MTT viability, indicating a pronounced reduction in metabolic viability and leaving insufficient viable/metabolically active cells for subsequent molecular analyses. Therefore, lower concentrations were evaluated for combined treatment. Concentrations of PA and OA that individually resulted in approximately 70% MTT viability were combined to identify a condition that maintained sufficient metabolic viability while providing a measurable cellular response. Among the combinations tested, 0.4 mM PA + 0.8 mM OA resulted in approximately 50% MTT viability, whereas increasing the concentrations to 0.6/0.9 mM and 0.8/1.0 mM PA/OA reduced MTT viability to approximately 30% and 20%, respectively (Figure 2). Accordingly, 0.4 mM PA + 0.8 mM OA was selected as the combined treatment condition for subsequent experiments.

Taken together, these findings established the 24‐h treatment period and the experimental concentrations used to investigate fatty‐acid‐associated molecular responses to PA, OA, and combined PA/OA exposure in HepG2 cells.

### 3.2 Effect of Palmitate, Oleate, and Their Combination on Lipid Droplet Accumulation in HepG2 Cells

Intracellular lipid droplet accumulation was evaluated by Oil Red O staining following treatment of HepG2 cells with palmitate, oleate, and their combination (Pal&Olea). Representative microscopic images are shown in Figure 1I. Compared with the negative control, treatment with palmitate, oleate, and particularly their combination resulted in a marked increase in intracellular lipid droplet formation.

**FIGURE 1:**
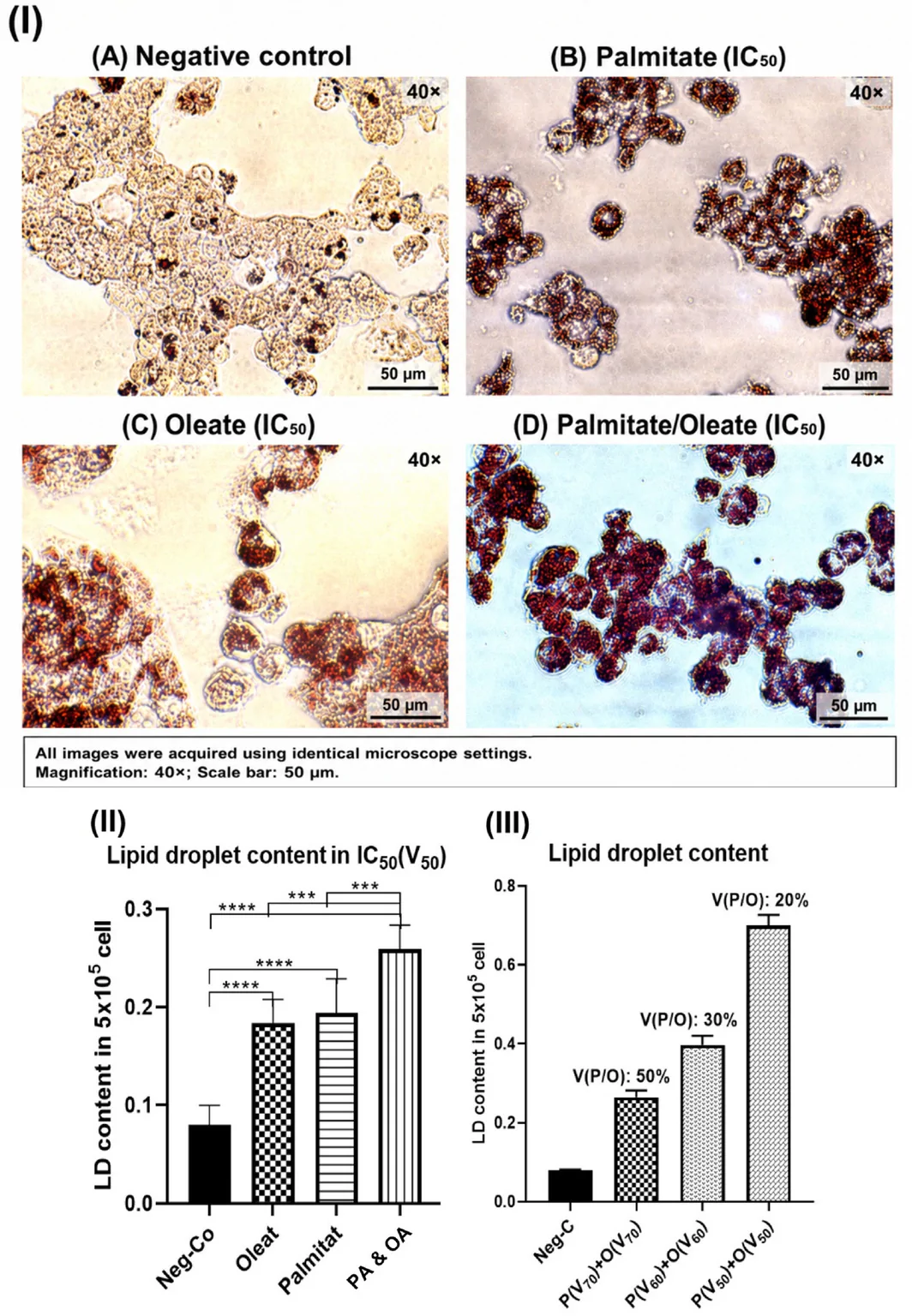
(I) Representative microscopic images (40× magnification) showing lipid droplet accumulation in HepG2 cells treated with the IC_50_ concentrations of palmitate (PA), oleate (OA), and palmitate/oleate (PA & OA). (II) Quantification of lipid droplet (LD) content in cells treated with the IC_50_ concentrations of palmitate, oleate, and PA&OA. (III) LD content in HepG2 cells treated with increasing combined concentrations of palmitate and oleate corresponding to V_70_, V_60_, and V_50_ conditions, demonstrating a dose‐dependent increase in lipid droplet accumulation (*p < 0.05; **p < 0.01; ***p < 0.001; ****p < 0.0001).

Semi‐quantitative analysis of Oil Red O staining demonstrated that lipid droplet content was significantly increased in all treated groups compared with the untreated control (*p* < 0.0001; Figure 1II). Among the individual fatty acid treatments, palmitate and oleate induced comparable levels of lipid accumulation, whereas the combined palmitate/oleate treatment (PA and OA) produced the highest lipid droplet content, indicating a stronger steatotic effect than either fatty acid alone.

To further evaluate the effect of combined fatty acid concentrations, HepG2 cells were treated with increasing concentrations of palmitate and oleate corresponding to the P(V_70_) + O(V_70_), P(V_60_) + O(V_60_), and P(V_50_) + O(V_50_) conditions. As shown in Figure 1III, lipid droplet accumulation increased progressively with increasing combined fatty acid concentrations. Lipid droplet content increased from approximately 0.25 in the P(V_70_) + O(V_70_) group to 0.40 in the P(V_60_) + O(V_60_) group and reached approximately 0.70 in the P(V_50_) + O(V_50_) group (*p* < 0.0001).

An inverse relationship was observed between cell viability and intracellular lipid droplet accumulation, with higher palmitate and oleate concentrations associated with greater lipid deposition and lower cell viability. Based on these findings, the IC_50_ concentrations of palmitate, oleate, and their combination were selected for subsequent experiments.

### 3.3 Effects of Palmitate, Oleate, and Their Combination on the Expression of WNT/β‐Catenin Signaling Components in HepG2 Cells

The effects of palmitate, oleate, and their combination (PA and OA) on the expression of key genes involved in the WNT/β‐catenin signaling pathway were evaluated in HepG2 cells. Distinct regulatory patterns were observed among the investigated genes, indicating differential modulation of the pathway by saturated and unsaturated fatty acids.

Palmitate significantly upregulated WNT expression (1.53‐fold, *p* = 0.0253), whereas oleate slightly reduced its expression without reaching statistical significance (Figure 2A). The combined treatment partially attenuated the stimulatory effect of palmitate on WNT expression, resulting in a nonsignificant increase relative to the control group. Consistent with WNT induction, palmitate significantly increased the expression of the downstream signaling components β‐catenin, TCF, and Axin2. β‐catenin expression was markedly elevated in all fatty acid‐treated groups, with the strongest response observed in palmitate‐treated cells (3.60‐fold increase, *p* < 0.0001) (Figure 2B). Similarly, TCF expression was significantly increased by palmitate (1.63‐fold, *p* < 0.0001) and by the combined treatment (1.42‐fold, *p* = 0.0029), whereas oleate had no significant effect (Figure 2D). Axin2, a canonical target gene of WNT/β‐catenin signaling, was significantly upregulated by palmitate (3.12‐fold, *p* = 0.0001) and by the combined treatment (2.60‐fold, *p* = 0.0008) (Figure 2G), while the increase induced by oleate did not reach statistical significance.

**FIGURE 2:**
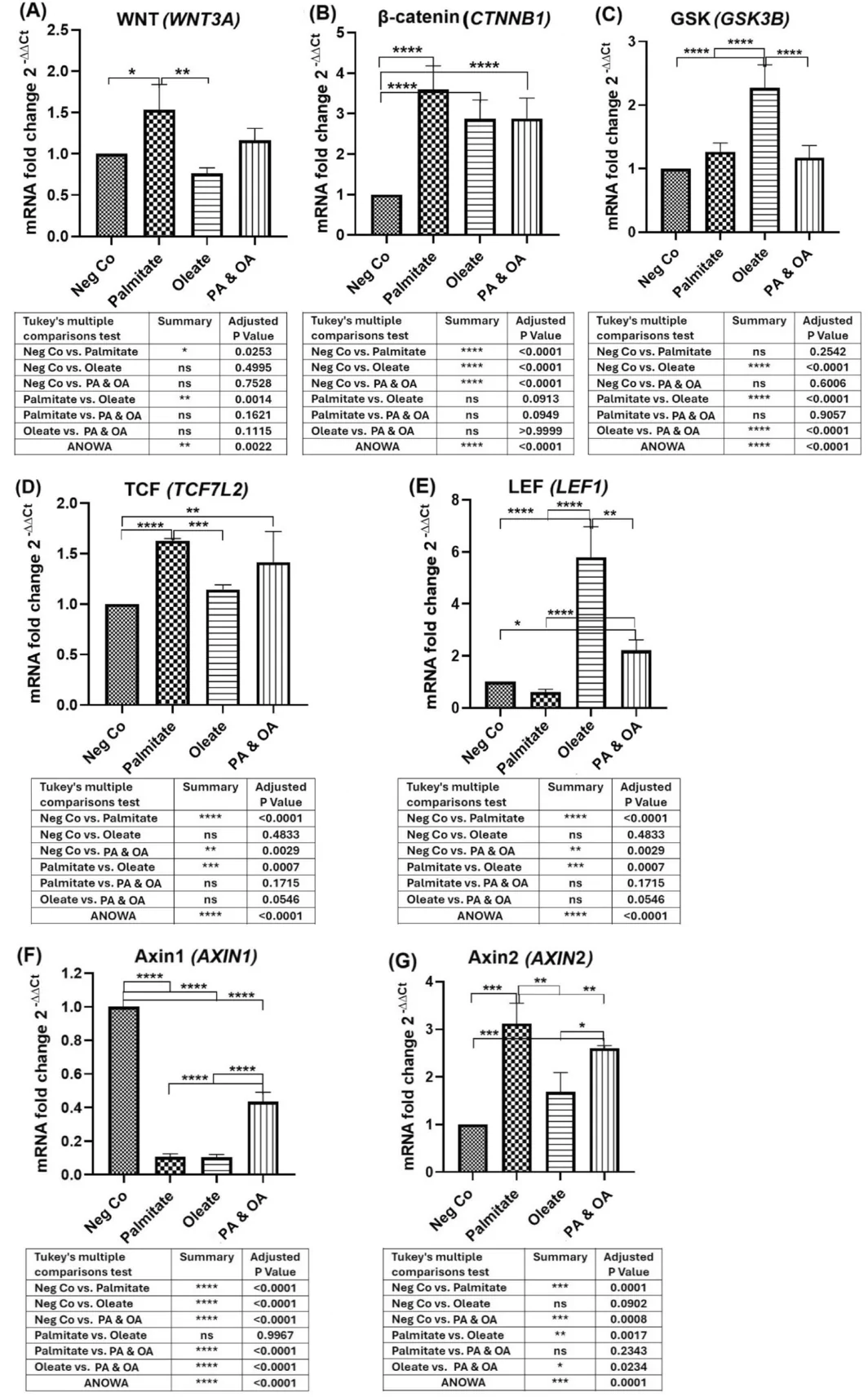
Transcriptional alterations in WNT (*WNT3A*), β‐catenin (*CTNNB1*), GSK (*GSK3B*), TCF (*TCF7L2*), LEF (*LEF1*), Axin1 (AXIN1), and Axin2 (*AXIN2*) were evaluated by RT‐qPCR following 24 h of exposure to palmitate (PA), oleate (OA), or combined palmitate/oleate (PA & OA). Target gene expression levels were normalized to the reference gene TBP and calculated using the 2^−ΔΔCt^ method, expressed as relative fold change compared with the Negative Control (Neg Co). Data are presented as mean ± SD from 2 to 4 independent experiments, as applicable. Statistical significance is indicated as follows: *p < 0.05; **p < 0.01; ***p < 0.001; ****p < 0.0001; ns, not significant.

In contrast, oleate exerted a distinct regulatory effect on other pathway components. LEF expression was strongly induced by oleate (5.79‐fold, *p* < 0.0001), representing the highest transcriptional response among all examined genes (Figure 2E). Although the combined treatment also significantly increased LEF expression (2.22‐fold, *p* = 0.0326) (Figure 2. E), the magnitude of induction was substantially lower than that observed with oleate alone. Likewise, GSK‐3β expression was significantly elevated only in the oleate‐treated group (2.27‐fold, *p* < 0.0001) (Figure 2C), whereas palmitate and the combined treatment produced no significant changes compared with the control group.

Analysis of Axin1 expression revealed a marked suppression by both palmitate and oleate, reducing transcript levels by approximately 89% relative to the control group (*p* < 0.0001 for both) (Figure 2F). Although Axin1 expression remained significantly lower than control levels in the combined treatment group, its expression was significantly higher than in cells treated with either fatty acid alone, suggesting a partial recovery effect following co‐treatment.

Overall, palmitate predominantly activated genes associated with canonical WNT/β‐catenin signaling, including WNT, TCF, β‐catenin, and Axin2, whereas oleate preferentially enhanced LEF and GSK‐3β expression. Co‐treatment with palmitate and oleate generally produced intermediate responses, attenuating several of the effects induced by each fatty acid alone while maintaining significant activation of selected WNT/β‐catenin pathway components.

### 3.4 Effects of Fatty Acids on WNT, β‐Catenin, and Glypican‐3 Protein Expression in HepG2 Cells

#### 3.4.1 WNT‐1 Protein Expression

Palmitate treatment significantly increased WNT‐1 protein expression compared with the control group (1.30‐fold, *p* < 0.05). This finding was consistent with the observed upregulation of WNT mRNA expression (1.53‐fold, *p* < 0.05). In contrast, oleate treatment reduced both WNT transcript and WNT‐1 protein levels, although neither change reached statistical significance. Co‐treatment with palmitate and oleate resulted in intermediate WNT mRNA and WNT‐1 protein expression levels, which were lower than those observed in the palmitate‐treated group. These results indicate that palmitate promotes WNT expression at both the transcriptional and protein levels, whereas oleate partially attenuates this response (Figure 3A).

**FIGURE 3:**
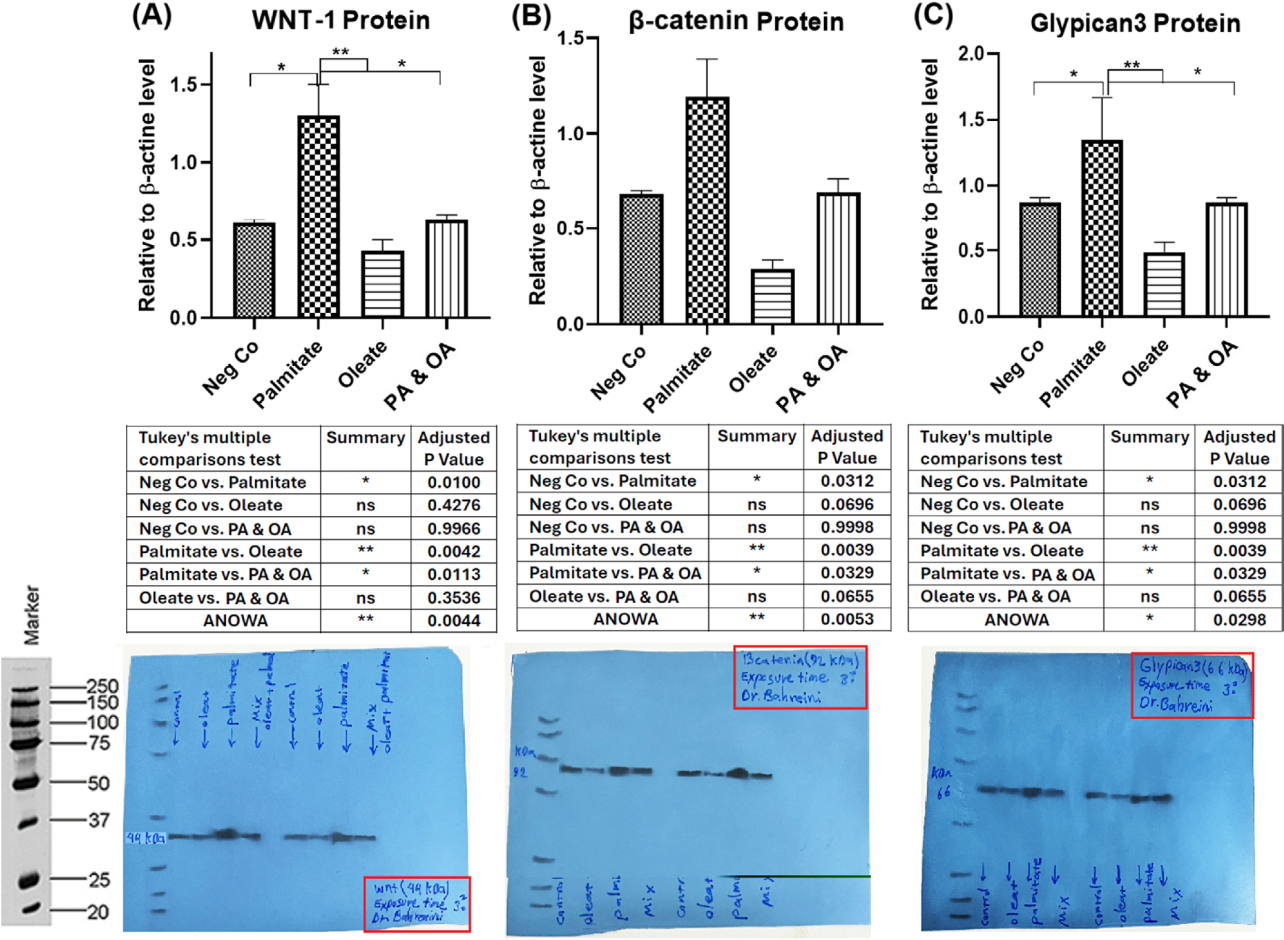
Quantitative densitometric analysis and representative Western blot bands for (A) WNT, (B) β‐catenin, and (C) Glypican‐3 (GPC3) protein levels following 24 h of exposure to palmitate, oleate, or combined palmitate/oleate (PA & OA). Protein expression levels were normalized to β‐Actin as the internal loading control. Data are presented as mean ± SD from 2 independent experiments, as applicable. Statistical significance is indicated as follows: *p < 0.05; **p < 0.01; ***p < 0.001; ****p < 0.0001; ns, not significant.

#### 3.4.2 β‐Catenin Protein Expression

Palmitate significantly increased β‐catenin protein expression relative to the control group (1.19‐fold, *p* < 0.05). This increase was accompanied by a marked elevation in β‐catenin mRNA expression (3.6‐fold, *p* < 0.0001). Oleate and the combined palmitate/oleate treatment also significantly increased β‐catenin transcript levels (2.87‐fold and 2.88‐fold, respectively; *p* < 0.0001), whereas no corresponding increase was observed at the protein level. β‐Catenin protein abundance in these groups remained comparable to, or slightly lower than, that of the control group. Thus, although all fatty acid treatments enhanced β‐catenin gene expression, only palmitate induced a significant accumulation of β‐catenin protein (Figure 3B).

#### 3.4.3 Glypican‐3 Protein Expression

Palmitate significantly elevated Glypican‐3 (GPC3) protein expression compared with the control group (1.35‐fold, *p* < 0.05). In contrast, oleate treatment resulted in a reduction in GPC3 protein levels, although this decrease was not statistically significant. Similarly, GPC3 expression in the combined‐treatment group did not differ significantly from that of the control group. Furthermore, GPC3 protein abundance was significantly higher in palmitate‐treated cells than in oleate‐treated or co‐treated cells. These findings demonstrate that palmitate promotes GPC3 protein expression, whereas the presence of oleate attenuates this effect (Figure 3C).

## 4 Discussion

The present study demonstrates that palmitate (PA) and oleate (OA) exert distinct effects on WNT/β‐catenin pathway‐associated molecular components in HepG2 cells under steatotic conditions. Although both fatty acids increased intracellular lipid accumulation, their effects on WNT‐related signaling differed substantially. Palmitate induced a coordinated molecular pattern characterized by increased WNT, β‐catenin, TCF, and Axin2 transcripts, together with increased WNT and β‐catenin protein expression and marked suppression of Axin1. In contrast, oleate increased LEF and GSK‐3β expression without producing a comparable coordinated response across the canonical pathway. Co‐treatment with PA and OA generally resulted in intermediate molecular responses, with partial attenuation of several PA‐associated changes. However, because the PA concentration differed between the PA‐alone and combined‐treatment conditions, these differences cannot be interpreted as direct evidence of an oleate‐mediated protective or antagonistic effect. Rather, they indicate that combined fatty‐acid exposure produced a molecular response distinct from that observed with PA alone. These findings suggest that fatty acid composition may influence signaling responses in ways that are not fully explained by total lipid accumulation alone.

The lipid‐loading experiments confirmed progressive intracellular lipid accumulation with increasing fatty acid exposure, accompanied by reduced cell viability. This apparent coexistence of lipid storage and cellular injury is consistent with the concept that neutral lipid deposition is not, by itself, an indicator of cellular protection. Previous studies have proposed that hepatic lipid droplets may contribute to the sequestration of potentially harmful fatty acids into neutral lipid stores, although the capacity of this buffering response may be limited under conditions of fatty acid excess [32, 33, 34]. In particular, palmitate‐associated cellular injury has been linked in previous studies to alterations in bioactive lipid species, including ceramides and diacylglycerols, as well as to oxidative, endoplasmic reticulum, mitochondrial, and inflammatory stress [33]. However, these lipid species and cellular stress pathways were not directly assessed in the present study and therefore cannot be considered demonstrated mechanisms underlying the observed reduction in cell viability [32, 33, 34]. Conversely, oleate has been reported to promote neutral lipid and lipid‐droplet formation and, in some experimental settings, to reduce palmitate‐associated cellular stress [35]. These previous observations provide a possible biological context for the different responses to PA and OA observed in the present model, but the underlying metabolic and stress‐related mechanisms remain to be established experimentally. Thus, the increased lipid‐droplet content observed in the present model should be interpreted primarily as evidence of increased intracellular neutral lipid accumulation, rather than as direct evidence of enhanced triglyceride synthesis, reduced lipotoxicity, or activation of specific cellular stress pathways.

The most relevant finding of the present study concerns the differential regulation of WNT/β‐catenin pathway‐associated components by PA and OA. Palmitate produced a relatively coordinated molecular signature, with increased WNT, CTNNB1, TCF, and Axin2 expression and substantial reduction of Axin1 [36, 37]. This molecular profile is consistent with enhanced canonical WNT/β‐catenin signaling and agrees with previous reports demonstrating that saturated fatty acids promote oncogenic signaling under lipotoxic conditions [38, 39]. This finding is particularly relevant to HCC biology because aberrant WNT/β‐catenin signaling is an established molecular feature of HCC and has been implicated in cellular processes that contribute to tumor development and progression. Thus, the PA‐associated molecular changes observed in the present HCC‐derived model may represent a cancer‐relevant signaling response to saturated fatty acid exposure, rather than simply a molecular consequence of intracellular lipid accumulation. The concomitant increase in WNT and β‐catenin protein levels further supports this interpretation. Mechanistically, reduced Axin1 expression may favor β‐catenin stabilization because Axin1 is an important scaffold of the β‐catenin destruction complex [40], whereas increased Axin2 may reflect activation of a downstream transcriptional response and an accompanying negative‐feedback mechanism [41]. Therefore, the simultaneous suppression of Axin1 and induction of Axin2 is compatible with a shift toward enhanced canonical WNT/β‐catenin signaling in PA‐treated cells. This interpretation is also consistent with previous evidence linking saturated fatty acids and lipotoxic stress to dysregulated oncogenic signaling [38, 39]. Nevertheless, because nuclear localization and transcriptional activity of β‐catenin were not directly assessed, the present findings should be regarded as molecular evidence consistent with enhanced pathway signaling rather than definitive proof of functional pathway activation.

Oleate produced a qualitatively different molecular response. Although CTNNB1 mRNA was significantly increased, β‐catenin protein abundance was not, and neither WNT nor TCF showed significant induction. Moreover, Axin2 remained unchanged, arguing against a coordinated activation of the canonical pathway. The marked increase in GSK‐3β expression provides additional context, as GSK‐3β participates in β‐catenin phosphorylation and degradation, although its phosphorylation state and enzymatic activity were not evaluated in this study. Similarly, the pronounced induction of LEF by OA should not be interpreted as direct evidence of canonical WNT activation. TCF and LEF can participate in overlapping as well as context‐dependent transcriptional programs [42, 43]. and the present study assessed their expression only at the mRNA level. Collectively, the OA‐associated increase in β‐catenin transcript without corresponding protein accumulation or induction of established downstream markers suggests that transcriptional regulation alone is insufficient to infer sustained canonical WNT/β‐catenin activity. Posttranscriptional and posttranslational mechanisms may contribute to this dissociation between CTNNB1 mRNA and protein abundance [44, 45]. Accordingly, the OA‐associated molecular response appears less compatible with coordinated canonical WNT/β‐catenin signaling than the response observed following PA exposure. From an HCC‐related perspective, this distinction suggests that different fatty acid species may create distinct signaling environments in HCC‐derived cells despite producing a common phenotype of intracellular lipid accumulation.

An additional observation was the strong suppression of Axin1 by both individual fatty acids. Although reduced Axin1 expression would theoretically favor β‐catenin stabilization, increased β‐catenin protein was observed only following PA treatment. This finding emphasizes that regulation of the canonical WNT pathway depends on the integrated behavior of multiple components rather than on a single regulatory molecule. In this context, the distinct effects of PA and OA on WNT, β‐catenin, GSK‐3β, TCF/LEF, and Axin proteins suggest that fatty acid quality may influence the balance between pathway‐promoting and pathway‐restraining signals. Importantly, the combined PA/OA treatment partially attenuated PA‐induced WNT and Axin2 responses and partially restored Axin1 expression. The intermediate molecular phenotype observed with co‐treatment is therefore compatible with a differential response to combined fatty‐acid exposure rather than a simple additive effect. Given previous reports that oleate may favor neutral lipid storage and modulate cellular responses to palmitate [32, 33, 34], altered lipid handling represents one possible context for the observed molecular differences. However, this possibility was not directly tested in the present study, and the contribution of oleate to the combined response cannot be established from the current experimental design.

From a hepatocarcinogenesis perspective, these findings are potentially relevant because aberrant WNT/β‐catenin signaling is a well‐established contributor to HCC development [18, 19, 20]. The present data suggest that exposure to a saturated fatty acid can produce a molecular environment more compatible with enhanced WNT/β‐catenin signaling, whereas oleate does not generate the same coordinated pattern. Because the WNT/β‐catenin pathway regulates cellular processes relevant to HCC, including proliferation, survival, and differentiation, differential modulation of this pathway‐associated molecular network may be relevant to the biological behavior of hepatic cancer cells under conditions of lipid stress [46]. This distinction may be particularly relevant in metabolic liver disease, where hepatocytes are exposed to mixtures of fatty acids rather than to individual lipid species. In this context, the present findings provide a potential molecular link between fatty acid composition, hepatic lipotoxic stress, and cancer‐relevant WNT/β‐catenin‐associated signaling. However, the current findings should not be interpreted as evidence that PA directly initiates HCC or that OA prevents hepatocarcinogenesis. Rather, they identify a differential molecular response that may contribute to the biological consequences of distinct fatty acid compositions during hepatic lipotoxic stress and may be relevant to signaling processes associated with HCC biology.

Several limitations of the present study should be acknowledged. First, the study was performed exclusively in HepG2 cells, a HCC‐derived model with a transformed phenotype and potentially altered WNT/β‐catenin regulation. Accordingly, the findings cannot be directly extrapolated to normal hepatocytes or to the early stages of MASLD [47, 48, 49]. The absence of non‐transformed or primary human hepatocyte models and in vivo validation further limits the generalizability of the findings to normal hepatic physiology and whole‐organism responses. Second, functional WNT/β‐catenin activation was not directly assessed through β‐catenin nuclear localization, active/non‐phosphorylated β‐catenin measurement, or β‐catenin‐dependent transcriptional assays. In addition, GSK‐3β phosphorylation status and enzymatic activity were not evaluated. Therefore, the observed alterations in WNT/β‐catenin‐related components should be interpreted as molecular changes consistent with pathway modulation rather than definitive evidence of functional pathway activation. Future studies incorporating functional assays and more physiologically relevant hepatic models will be required to further validate these findings. Another limitation is that lipidomic and detailed metabolic analyses were not performed. Therefore, specific intracellular lipid species, oxidative stress responses, endoplasmic reticulum stress, mitochondrial function, inflammatory mediators, and related metabolic alterations were not directly evaluated and remain important areas for future investigation. Additionally, the MTT assay provides an indirect assessment of cellular metabolic activity/viability and cannot independently distinguish changes in metabolic activity from alterations in cell number or specific cell death mechanisms. A further limitation concerns the design of the combined PA/OA treatment. Because the PA concentration differed between the PA‐alone and combined‐treatment conditions, the present design does not allow the specific effect of adding OA to a fixed PA exposure to be determined. Thus, the lower magnitude of some molecular responses observed in the combined group cannot be conclusively attributed to an oleate‐mediated protective effect. Future studies using matched PA concentrations with and without OA, together with appropriate OA‐alone conditions, will be necessary to clarify the specific contribution and interaction of these fatty acids.

## 5 Conclusions

In conclusion, the present study demonstrates that palmitate and oleate differentially modulate molecular components associated with the canonical WNT/β‐catenin signaling pathway in lipid‐loaded HepG2 cells. Palmitate induced a coordinated molecular response characterized by increased expression of WNT3A, CTNNB1, TCF7L2, and AXIN2 transcripts, together with increased WNT and β‐catenin protein levels and reduced AXIN1 expression. In contrast, oleate produced a more selective response, characterized by alterations in individual pathway components without evidence of increased β‐catenin protein abundance or a coordinated pattern of canonical pathway activation. Combined palmitate/oleate exposure resulted in an intermediate molecular phenotype and partially attenuated several palmitate‐associated alterations. Overall, these findings indicate that palmitate and oleate differentially influence WNT/β‐catenin pathway‐associated molecular responses in lipid‐loaded HepG2 cells, suggesting that fatty acid composition may contribute to differences in cellular signaling responses during lipid overload. However, these findings represent indirect molecular evidence obtained from a single HCC‐derived in vitro model and require further validation using primary or non‐transformed hepatocyte models, direct functional assessment of WNT/β‐catenin activity, and appropriate in vivo approaches.

## Author Contributions

**Moayed Al‐Haeri:** investigation, methodology, data curation, writing – original draft, writing – review and editing. **Yaser Mohammadi:** conceptualization, methodology, validation, investigation, data curation, writing – review and editing, writing – original draft, formal analysis. **Roghiyeh Pashaei‐Asl:** investigation, formal analysis, data curation, writing – review and editing. **Elham Bahreini:** conceptualization, investigation, validation, visualization, software, methodology, formal analysis, data curation, supervision, writing – original draft, writing – review and editing.

## Funding

This project was supported by the Iran University of Medical Sciences (Grant No. 32924).

## Ethics Statement

The study model was approved by the Ministry of Health of Iran (Ethical code: IR.IUMS.REC.1403.1106).

## Conflicts of Interest

The authors declare no conflicts of interest.

## Data Availability

Data available on request from the authors.
